# Endoscopic ultrasound guided resection of a Cushing’s adenoma invading the medial cavernous sinus wall using the "interdural peeling" technique

**DOI:** 10.3171/2023.4.FOCVID22150

**Published:** 2023-07-01

**Authors:** Guilherme Finger, Kyle C. Wu, Jaskaran S. Gosal, Basit Jawad, Joshua Vignolles-Jeong, Ricardo L. Carrau, Daniel M. Prevedello

**Affiliations:** Departments of 1 Neurosurgery and; 2 Otolaryngology, The Ohio State University Wexner Medical Center, Columbus; and; 3 The Ohio State University College of Medicine, The Ohio State University, Columbus, Ohio

**Keywords:** Cushing’s adenoma, endoscopic ultrasound, endoscopic endonasal approach, medial cavernous sinus wall

## Abstract

Cushing’s adenoma invading the cavernous sinus requires aggressive resection to be cured. MRI is frequently inconclusive for identifying microadenomas, and visualizing the involvement of the medial cavernous sinus is even more challenging. In this video, the authors present a patient with an adrenocorticotropic hormone (ACTH)–producing microadenoma with doubtful left medial cavernous sinus involvement on MRI. She underwent an endoscopic endonasal exploration of the medial compartment of the cavernous sinus. The abnormally thickened wall, confirmed by intraoperative endoscopic endonasal ultrasound, was safely excised using the "interdural peeling" technique. Complete resection of the tumor resulted in normalization of her postoperative cortisol levels and disease remission with no complications.

The video can be found here: https://stream.cadmore.media/r10.3171/2023.4.FOCVID22150

## Transcript

### 0:21 Introduction and Patient Details.

This video demonstrates an endoscopic endonasal transsellar and transcavernous approach for resection of a pituitary adenoma. It was invading the medial wall of the cavernous sinus. The patient had a confirmation of Cushing’s disease and an MRI demonstrating a hypointensity on the left side of the sella that was questionable for invasion of the medial wall of the cavernous sinus and the medial compartment of the cavernous sinus.

### 0:48 Initial Exposure.

Patient was brought to the operating room and an endoscopic endonasal approach was performed. Binostril approach was accomplished and the sella was drilled in a standard fashion.

### 0:56 Drilling of the Sella and Opening of Dura.

Our initial plan was to explore the sella and evaluate the medial wall of the cavernous sinus to then make a decision if a cavernous sinus approach would be needed.^1^ We drill the sella, initially exposing the dura of the sella. The dura of the sella was opened sharply with a blade.

### 1:24 Opening of the Cavernous Sinus Using Interdural Peeling Technique.

The subdural compartment was dissected, separating the dura from the tunica of the pituitary gland. With endoscopic scissors, we were able to expand the opening particularly on the left side, where the disease was located. Initial dissection demonstrated just a pituitary gland in the area. We explored the medial aspect of the medial wall of the cavernous sinus.^2^ We prepared for potential dissection in the direction of the cavernous sinus by dividing the space between the periosteal and meningeal layers of the dura of the face of the sella.

### 1:41 Extracapsular Dissection of Pituitary Adenoma.

At this point, we didn’t see any obvious invasion in that compartment, and we felt that potentially it would be just a pituitary adenoma located in the sella. We dissect the pituitary adenoma in extracapsular fashion, identified the structures, and confirmed with an ultrasound.

### 2:29 Use of Endoscopic Endonasal Ultrasound.

With the ultrasound we were able to see the pituitary adenoma located superficially and laterally on the left side; also, a clear-cut separation between the pituitary gland anterior portion and the posterior portion of the pituitary gland. We could also see the carotid artery more laterally.^3–5^

### 2:58 Continued Dissection and Adenoma Removal From Posterior Pituitary Gland.

We continued our dissection, and we were able to obtain an extracapsular resection of the pituitary adenoma that was located on the left side of the pituitary gland. We were very careful not to damage the posterior gland to avoid diabetes insipidus, and we dissected posteriorly, and the ultrasound during the surgery was also important to define that we were not reaching the posterior gland. We dissected away from the posterior gland, and we were able to rotate the pituitary adenoma from the sella.

### 3:38

There was some attachment to the lateral wall that made us worried that this was infiltrating the medial wall of the cavernous sinus. At this point we removed the pituitary adenoma, carefully watching the behavior in relation to the medial wall of the cavernous sinus.

### 4:09 Start of Medial Cavernous Sinus Wall Exploration.

We were able to remove, and we felt that there was some thickness located laterally in the medial wall of the cavernous sinus, so we went back to that space and analyzed, and when we elevate the medial wall of the cavernous sinus it looked extremely thick. And we then confirmed the need for expansion. And we then opened the space by drilling the anterior wall of the cavernous sinus, exposing the periosteum of the carotid artery on the left side.^6^ With more space, we’re able to cut the ligaments located inside the cavernous sinus and rotate the medial wall of the cavernous sinus away.

### 4:42 Continued Dissection and Removal of the Abnormal Medial Wall of the Cavernous Sinus.

And initially we cut the inferior portion of the medial wall of the cavernous sinus that was extremely thick and protecting always the carotid artery that was located more laterally. We remove the first piece of the medial wall of the cavernous sinus, and send that to pathology. And we were still seeing some thickening superiorly; what you see immediately lateral, that’s the carotid artery, so there was no obvious tumor located inside the cavernous sinus, but the wall definitely look thicker than usual. So we expanded the opening superiorly to allow us to do the final cut in the abnormal medial wall of the cavernous sinus without causing any CSF leak or causing any problems with the cavernous sinus structures. We were able to remove the last piece that was abnormal of the medial wall of the cavernous sinus.

### 5:35 Reconstruction.

We then irrigated with hydrogen peroxide and performed reconstruction with collagen matrix, followed by the mucosa of the sphenoid sinus that was rotated back in place, covering the sella and the entire skull base defect.

### 5:50 Postoperative Course and Recovery.

Patient did very well after surgery. We noticed a peak of ACTH that happened intraoperatively, followed by the descent of ACTH to undetectable levels. The cortisol was measured every 6 hours and proved to go down below normality.

### 6:08 Conclusions.

In conclusion, we prove that the ultrasound was a very useful tool to identify the microadenoma, particularly immediately medial to the internal carotid artery, and the interdural peeling was a great method to enter the cavernous sinus.

## Disclosures

Dr. Prevedello reported consulting for Stryker, Medtronic, BK Medical, and Integra LifeSciences; and royalties from KLS Martin, ACE Medical, and Mizuho.

## Author Contributions

Primary surgeon: Prevedello. Assistant surgeon: Wu, Jawad, Carrau. Editing and drafting the video and abstract: all authors. Critically revising the work: Finger, Wu, Gosal, Jawad, Carrau, Prevedello. Reviewed submitted version of the work: Finger, Wu, Jawad, Carrau, Prevedello. Approved the final version of the work on behalf of all authors: Finger. Supervision: Wu, Prevedello.

## Supplemental Information

### Patient Informed Consent

The necessary patient informed consent was obtained in this study.
